# Physiological effects of oral glucosamine on joint health: current status and consensus on future research priorities

**DOI:** 10.1186/1756-0500-6-115

**Published:** 2013-03-26

**Authors:** Yves Henrotin, Xavier Chevalier, Gabriel Herrero-Beaumont, Timothy McAlindon, Ali Mobasheri, Karel Pavelka, Christiane Schön, Harrie Weinans, Hans Biesalski

**Affiliations:** 1 Bone and Cartilage Research Unit, University of Liège, Institute of Pathology, Level +5, CHU Sart-Tilman, Liège, 4000, Belgium; 2 Princess Paola Hospital, Marche-en-Famenne, Belgium; 3 Rheumatology Department, Mondor Hospital, Créteil, France; 4 Bone and Joint Research Unit, Service of Rheumatology, Fundación Jiménez Díaz, Universidad Autónoma, Madrid, Spain; 5 Division of Rheumatology, Tufts Medical Center, 800 Washington Street, Box 406, Boston, MA, 02111, USA; 6 Musculoskeletal Research Group, Division of Veterinary Medicine, School of Veterinary Medicine and Science, Faculty of Medicine and Health Sciences, The University of Nottingham, Sutton Bonington Campus, Sutton Bonington, Leicestershire, LE12 5RD, United Kingdom; 7 Institute of Rheumatology, Prague, Czech Republic; 8 UMC Utrecht, Department of Orthopedics, Heidelberglaan 100, Utrecht, 3584 CX, The Netherlands; 9 Erasmus University Medical Center, Rotterdam, The Netherlands; 10 Department of Biological Chemistry and Nutrition, Garbenstrasse 30, Stuttgart, D 70593, Germany

**Keywords:** Glucosamine, Osteoarthritis, Treatment, Prevention

## Abstract

The aim of this paper was to provide an overview of the current knowledge and understanding of the potential beneficial physiological effects of glucosamine (GlcN) on joint health. The objective was to reach a consensus on four critical questions and to provide recommendations for future research priorities. To this end, nine scientists from Europe and the United States were selected according to their expertise in this particular field and were invited to participate in the Hohenheim conference held in August 2011. Each expert was asked to address a question that had previously been posed by the chairman of the conference. Based on a systematic review of the literature and the collection of recent data, the experts documented the effects of GlcN on cartilage ageing, metabolic/kinetic and maintenance of joint health as well as reduction of risk of OA development. After extensive debate and discussion the expert panel addressed each question and a general consensus statement was developed, agreeing on the current state-of-the-art and future areas for basic and clinical studies. This paper summarizes the available evidence for beneficial effects of GlcN on joint health and proposes new insight into the design of future clinical trials aimed at identifying beneficial physiological effect of GlcN on joint tissues.

## Discussion

Glucosamine (GlcN) may be a suitable intervention for osteoarthritis (OA) and, indeed, is already self-administered by millions of individuals without any apparent deleterious side-effects. GlcN is and ubiquitous amino sugar that is believed to play a key role in cartilage formation and repair. It is a naturally occurring substance in the body and is essential for the normal growth and repair of connective tissues such as articular cartilage in synovial joints. GlcN is one of the major constituents of the extracellular matrix (ECM) of cartilage and a building block for proteoglycan synthesis. It exists in several forms, GlcN sulfate (GlcN.S) and GlcN hydrochloride (GlcN.H) being the most widely used and studied. GlcN.S has been reported to be efficient on knee OA symptoms [1-5].

In this paper, our goal was to explore new avenues concerning the use of GlcN, especially in terms of beneficial and disease preventing physiological effects, notably with respect to OA development. To address this aim, eight experts from six European countries and one expert from the United States were selected and invited according to their expertise in the context of this particular field, to the Hohenheim conference, which was held in August 2011. Each expert was asked to address a question that has been previously posed by the chairman of the conference. Responses were based on review of the published literature and the collection of the most relevant papers and recent relevant data. Following extensive debate and discussion, a general consensus was reached in relation to each question and new strategies were proposed and discussed for basic and clinical future works (Tables 1 and 2).

**Table 1:** Summary of the experts group consensus

	
1	At the present time there is no evidence that glucosamine modulates ageing phenotype and no studies have been performed on the effects of glucosamine on aged cartilage.
2	New and more focused studies are clearly needed to determine if GlcN can affect oxidative stress, mitochondrial function, autophagy and responsiveness to cytokines and growth factors.
3	Chondrocytes have the capacity to biosynthesize glucosamine from glucose in health and disease. There is no evidence that chondrocyte glucosamine requirement is modified with age or pathological situation.
4	The endogenous level of glucosamine is comprised between 1μM and 2μM, and reach to 10 μM after oral administration of a therapeutic dose (1500 mg);
5	There is no information about the optimal dose use and no information of the interference of diet, age and other gastrointestinal comorbidities or association with other nutraceuticals most particularly with chondroitin sulfate on the GlcN phamarcokinetic profile.
6	Glucosamine might contribute to the maintenance of healthy joints, but this should be confirmed in clinical trials designed to investigate its effects on healthy subjects with high risk of osteoarthritis and using parameters, which establish the link between consumption of glucosamine and maintenance of joints (e.g. biochemical markers and/or MRI).
7	In vitro and ex vivo studies on normal and OA chondrocytes demonstrated stimulating effect of GlcN with supra-pharmacological concentrations on the synthesis of cartilage matrix components and inhibiting potencies on pro-catabolic and pro-inflammatory factors.
8	Prophylactic evidence has been shown in some animal models, but not in human.
9	More research is necessary to explore possible beneficial effects of GlcN in healthy subjects or on risk factors of OA.

**Table 2:** Consensus and research recommendations for each question addressed

**Question 1. What is the main impact of cartilage ageing? Is GlcN effective against cartilage ageing?**	
**Consensus**	**Research recommendations**
Although older age is the greatest risk factor for OA, OA is not an inevitable consequence of growing old.	To investigate GlcN on the following parameters:
	• Telomere erosion
1. Chondrocytes have the capacity to biosynthesize GlcN from glucose in health and disease. There is no evidence that chondrocyte GlcN requirement is modified with age or pathological situation.	• Mitochondrial dysfunction
	• Reactive oxygen species (ROS) and antioxidants production
2. To study the influence of ageing on the synthesis of GlcN by chondrocytes in *in vitro* and spontaneous models of OA in animal is recommended.	• Responsiveness to anabolic growth factors
3. Study of the production of GlcN by chondrocytes at different stages of OA development in animal models should be performed.	• Autophagy
4. The senescence-associated secretory phenotype of articular chondrocytes should be better characterized to differentiate ageing and OA affected cartilage.	• Apoptosis
5. At the present time there is no evidence that GlcN modulates this phenotype and no studies have been performed on the effects of GlcN on aged cartilage. New and more focused studies are clearly needed to determine if GlcN can affect oxidative stress, mitochondrial function, autophagy and responsiveness to cytokines and growth factors.	• The expression of transcription factors associated with longevity (i.e. SirT1 and FoxO)
6. There is some in vitro evidence that GlcN modulate glucose metabolism is chondrocytes.	• The accumulation Advanced Glycation End products in ECM.
7. There is some evidence on systemic effect of GlcN through reduction of inflammatory marker and anabolic effects on cartilage.	
**Question 2. How does the metabolic kinetic look like? What about the Cmax levels?**	
**Consensus**	**Research recommendations**
1. Heterogeneous level of endogenous level of GlcN is comprised between 1μM and 2μM	• To study the influence of various diets enriched in GlcN and ageing on the endogenous level of GlcN
2. Translation of animal models concerning the basal level must been done with caution because there are species differences in GlcN in absorption and bioavailability.	• To better quantify the resting plasma levels of GlcN with future developments in MS-technologies and bioanalytical techniques that should contribute to the standardization of assays used to detect.
3. There is no information on the interference of diet, age and other gastrointestinal comorbidities on the GlcN phamarcokinetic profile.	• To study higher doses of GlcN in human OA to look for beneficial mechanisms of action of GlcN.S in experimental models
	• To perform more pharmacokinetic and pharmacodynamic studies in human as well as in animals
	• To consider the formulation and complexation of the preparations in future *in vitro* studies on GlcN
	• To take into account the serum and synovium levels from clinical trials (physiological effects found around serum levels of 10μM) to determine the *in vitro* concentration used in future studies should take into account
	• To better investigate the distribution of GlcN in the different joint tissues
**Question 3. How can GlcN contribute to cartilage maintenance in healthy subjects, and how can it be demonstrated?**	
**Consensus**	**Research recommendations**
1. GlcN might contribute to the maintenance of joint health in OA patients, but this should be demonstrated in clinical trials designed to investigate its effect on healthy subjects with high risk of OA and evaluating parameters which can establish the link between consumption of GlcN and the maintenance of joints (e.g. biochemical markers and/or MRI). This statement is based on the following observations:	• To investigate the effect of GlcN in the maintenance of healthy subjects. This should be done as recommended in the framework of OARSI, in young subjects with high risk factors of OA
	• To investigate the effects of GlcN on a panel of functional imaging and biochemical markers investigating joint tissue metabolism.
- *In vitro* and *ex vivo* studies on normal and OA chondrocytes demonstrated stimulating effect of GlcN with supra-pharmacological concentrations on the synthesis of cartilage matrix components and inhibiting potencies on pro-catabolic and pro-inflammatory factors.	
- Prophylactic evidence has been shown in some animal models.	
- Up-to-date, there is no convincing data demonstrating the potential benefit of GlcN in healthy subjects.	
**Question 4. Can GlcN reduce the risk of OA development?**	
**Consensus**	**Research recommendations**
1. No data on modulating risk factors of OA.	• To search the influence of GlcN on metabolic (e.g. adipokine secretions, systemic inflammation) and structural risk factors (e.g.; osteophytes, bone marrow lesions)
2. Some animal and cases report in human suggest that chronic use of GLcN in diabetes or “pre-diabetes” individuals might lead to insulin resistance. However the level of proofs is not sufficient and we lack long term studies of GlcN use for individuals with diabetes or pre diabetes. Based on the available evidence, no specific recommendation for controlling glycemia in patients taking GlcN could be addressed	• To identify surrogate marker for risk factors investigation (e.g. multiplex biological test including markers of inflammation, glucose, fat tissue, bone and cartilage metabolism or aggregate score integrating biological, imaging and clinical)
	• To design clinical trials on well-defined subgroups with a specific risk profile (metabolic syndrome, etc.) to demonstrate beneficial effects of GlcN on risk factors or risk profile.

This paper addresses some questions related to the evidence on the beneficial effects of GlcN on the joint health. Based on a research of the literature performed on PubMed, the experts attempted to document the effects of GlcN on cartilage ageing and maintenance of joint health, two key points for the substantiation of health claims. They have also provided some suggestions for the design of future clinical trials in order to identify beneficial physiological effects of GlcN on joint tissues.

### What is the main impact of cartilage ageing? is GlcN effective against cartilage ageing?

Ageing is a major contributor to musculoskeletal impairment, the degeneration of joint tissues and the development of OA [6,7]. Age-related changes in articular cartilage contribute to the development and progression of OA. Although the degeneration of articular cartilage is not simply the result of ageing and mechanical wear, it nevertheless modifies the articular joint tissues and synovial fluid [6,8]. Although older age is the greatest risk factor for OA, OA is not an inevitable consequence of growing old [9]. The mechanisms for the link between ageing and OA are incompletely understood. Cell stress and oxidative damage contribute to chronic inflammation that promotes age-related diseases. Ageing chondrocytes express a so-called “senescent” secretory phenotype, which has some common characteristics with chondrocyte phenotype in OA, especially in relation to the production and secretion of pro-inflammatory cytokines, chemokines, and proteases [10].

One may question if the oral intake of GlcN could interfere with chondrocyte senescence mechanisms. At this time, it remains difficult to answer the question “is GlcN effective against cartilage ageing or is this compound effective on the ageing phenotype”. Indeed there is no published evidence to suggest that GlcN is effective against cartilage or any other connective tissues ageing. Although there is some evidence to suggest that glucose can accelerate *in vitro* cell senescence, there is no evidence that GLcN can act in one way or another on it. However, considerable evidence for indirect effects of GlcN on ageing has been obtained. A recent proteomic study [11] has shown that GlcN.S alters the expression of proteins involved in signal transduction pathways, redox and stress responses, and protein synthesis and folding processes in human chondrocytes. Interestingly, GlcN affects mainly energy production and metabolic pathways. However, there is no “mechanistic” data linking GlcN.S to the reduction of oxidative stress in *in vitro* or animal studies.

Another study suggests that alpha-tocopherol, ascorbic acid, selenium, combined GlcN.S and chondroitin exert antioxidant effects on cultured chondrocytes [12]. However, this study used combination products. It is therefore difficult to determine whether GLcN by itself has any direct and specific effects on oxidative stress.

Finally, a study investigated the effects of GlcN.S on heme oxygenase (HO-1), p22 (Phox) (a subunit of NADPH complex) and inducible nitric oxide synthase (iNOS) expression by primary human chondrocytes *in vitro*[13]. HO-1 gene expression was up-regulated by 10 mmol/l GlcN.S (24 h). Expression of the p22 (Phox) gene was down-regulated by 10 mmol/l GlcN.S (48 h treatment); the HO-1 gene was down-regulated by IL-1β and 10 mmol/l GlcN.S appeared to restore the expression levels to baseline values.

It has recently been described that OA was associated with an alteration in the O-N-actetyl-glycosaminylation (O-GlcNAc) of proteins in the cartilage of patients [14]. Since the discovery of this protein modification by Torres and Hart [15], O-GlcNAc signalling has been implicated in a diverse array of physiological and pathological functions [16]. Furthermore, there is a growing recognition that global protein O-GlcNAc alterations are involved in the pathophysiology of chronic- and age-related diseases, such as diabetes, cardiac hypertrophy and failure, immunological disorders, cardiovascular disease, neurodegeneration and cancer [17,18]. In this sense, GlcN has been described to increase the flux through the hexosamine pathway modifying the amount of O-GlcNAc acylated proteins, and thus acting as a protective agent against cell stress and promoting cell survival [19]. Preliminary data from Herrero Beaumont’s laboratory show that glucosamine treatment could diminish OA damage in parallel with a diminution in O-GlcNAc modified proteins (Largo, unpublished data).

Another indirect effect of GlcN on ageing could happen through its effect on inflammation. A number of recent publications described anti-inflammatory effects of high dose of GlcN in rheumatoid arthritis models [20,21]. These findings are underlined by studies with physiological concentrations of GlcN.S where increased levels of mRNA and aggrecan core protein and a decrease of the production of MMP-3 in human OA chondrocytes were investigated [22]. The study of Byron et al. using equine chondrocytes and synovial cells found that GlcN.H decreased the IL-1-stimulated production of prostaglandins (PGEs) in both cell types [23].

### Do GLcN interfere with glucose metabolism in chondrocytes?

A key question is “is GlcN capable to interfere with glucose metabolism of chondrocytes?”. Chondrocytes are highly glycolytic and glucose is an important metabolic fuel for fully differentiated chondrocytes both during postnatal development and in adult articular cartilage [24]. Glucose is also a common structural precursor for the synthesis of extracellular matrix glycosaminoglycans (GAGs) [24]. In addition, chondrocytes express the insulin receptor and are very sensitive to extracellular and intracellular glucose levels [25]. Healthy chondrocytes have a negative feedback loop for controlling the uptake of glucose by modulating the expression of glucose transporter-1 (GLUT-1), depending on extracellular glucose levels. Therefore, if extracellular glucose increases, chondrocytes induce the degradation of GLUT-1. However in OA chondrocytes, this feedback appears to be disturbed. Hence, under high glucose, GLUT-1 transporters cannot adequately be down regulated and intracellular glucose accumulation occurs with subsequent deleterious effects [26,27]. This is an extremely interesting phenomenon that might have major repercussions for the aged population where undetected diabetes (i.e. subclinical, mild) and early OA occur frequently together. This fact might explain a potential mechanism of action of GlcN that shares the GLUT transporter system with glucose. Thus high concentrations of GlcN.S and GlcN.H might potentially act as a competitive inhibitor for glucose uptake [28] and protect chondrocytes against high glucose. Intracellular competition may also occur at substrate level since glucose and GlcN are both phosphorylated by the same enzyme, glucokinase (hexokinase). However, in turn, phosphorylated GlcN is an allosteric inhibitor of glucokinase. The consequence of this is a negative feedback loop that blocks glycolysis [29,30]. Nevertheless, different forms of GlcN might act somehow differently since N-acetyl-GlcN is phosphorylated by a different enzyme preventing the intracellular competition with glucose [31]. It might be possible that GlcN can achieve both, activating insulin resistance and/or blocking glycolysis, depending on cell phenotype, disease status and specific form of the amino sugar.

### How does the metabolic kinetic look like? What about the Cmax levels?

GlcN is highly active when added to cell cultures or used in animal models at high concentrations [32]. While several studies have published information about the mechanism of action of GlcN, its pharmacokinetics has only been recently described. The lack of sensitive analytical methods capable of detecting the compound in biological fluids has been the primary limitation of pharmacokinetic studies. In addition, other major difficulties associated with the quantitative determination of GlcN in biological samples are low GlcN levels and the presence of many structurally related sugar molecules that can interfere with its analysis [33,34].

Persiani et al. described the complete pharmacokinetic profile of GlcN, after three oral doses (once daily, consecutive administration) of 1500 mg crystalline GlcN.S in healthy volunteers [35]. GlcN is bioavailable from orally administered crystalline GlcN.S with maximum plasma concentrations up to 100-fold higher than endogenous levels, in the 10 μM range after approximately 3 h post ingestion. Pharmacokinetic parameters indicate that it is eliminated with a half-life estimated of approximately 15h, thus supporting the once-daily dosing. Finally, the pharmacokinetics was linear in the dose range of 750–1500 mg of GlcN.S, but not clearly defined at higher doses [35].

Older techniques were not sensitive enough to monitor GlcN plasma concentrations giving erratic values. Previous human and animal studies failed to detect circulating endogenous GlcN.S [35-37] or GlcN.H [38] at concentrations ranging from as low as 10 to as high as 200 ng/ml. The potential pathophysiological significance of this variability should be further investigated. Repeated once-daily doses of around 20 mg/kg GlcN.S showed a similar pharmacokinetic profiles with a peak plasma concentration (Cmax) around 10 μM, while the GlcN concentration in synovial fluid was only 23.5% lower than that in plasma [39,40]. Therefore, according to published results, GlcN is well absorbed after repeated oral administration. Furthermore, taking into account other pharmacokinetic studies in humans, the Cmax after a once daily dosing of 20 mg/kg GlcN.S is about 10 μM [32].

The National Institutes of Health (NIH)-sponsored the GlcN hydrochloride (GlcN.H)/Chondroitin sulfate Arthritis Intervention Trial (GAIT), which was aimed at studying the effect of these two compounds on pain and function in knee OA patients. As part of this initiative, pharmacokinetic studies were undertaken. GlcN concentrations found in this study were much lower than those found in patients treated with GlcN.S 1500 mg. Thus, after a single-dose of GlcN.H 500 mg, the mean plasma Cmax was 3 μM in healthy volunteers. Moreover, the Cmax and the area under the curve of GlcN were even lower when the GlcN.H was given chronically in fractioned doses of 500 mg three times daily reaching a Cmax of 211 ng/mL [41]. The mean plasma Cmax was achieved after a single dosage of 1500 mg GlcN.H, given orally with 6 capsules of 250 mg each after a three-month treatment. In addition, the bioavailability of GlcN was decreased in patients who took the compound in combination with chondroitin sulfate [41].

Therefore a topical and crucial question is whether 10 μM GlcN has biological effects in *in vitro* systems, animal models, and in human subjects. Animal studies showing significant effects of GlcN not only at cartilage level in OA models but also in other systems were achieved at doses 15- and 20-fold greater than typical doses consumed by human subjects [42,43]. Most *in vitro* studies of GlcN.S and GlcN.H activity on joint tissue and chondrocytes have been performed in the 500 and 5000 μM range [32,44]. Some studies have also described some beneficial effects of GlcN.S and GlcN.H at *in vitro* concentrations around 50 μM and even as low as 10 μM [23,32]. Nonetheless, the majority of published papers report much higher GlcN doses leading to high concentrations, far from those achieved after the recommended dosing in human OA treatment. For this reason, more studies are required to assess the biological activity of 10 μM GlcN in order to gain mechanistic insight of its actions on OA joint tissues [43].

Finally several key questions remain unanswered. Is ageing related to a decrease of endogenous GlcN synthesis? Do changes in eating habits contribute to elevated requirements of exogenous GlcN? Is endogenous GlcN enough to supply chondrocyte metabolism? At the present time these questions remain unanswered. These points should be clarified in future studies to estimate whether administering GlcN is pertinent and whether GlcN provides beneficial effects on joint health.

### How can GlcN contribute to cartilage maintenance in healthy subjects and how can this be demonstrated?

There are currently no studies that have tested GlcN as a chondroprotective agent in a preventive context. Nevertheless, there is some indirect evidence from published trials, showing a possible role of GlcN as a preventive agent in the progression of knee OA. GlcN.S indeed prevented joint space narrowing (JSN) in two randomised, placebo controlled, double blind trials of 3-year duration in knee OA [1,2]. Several important points can be gleaned from these trials. The first one is the mild to moderate characteristics of the patient population at baseline. The effects observed in this population may therefore be extrapolated, with some caution, to the general population at risk for OA. The second point is that the subgroup analysis of postmenopausal female patients showed more effects on joint structure in this subgroup than in the overall population [45]. Being a female carries a greater risk factor for the development of OA and the effects observed in this subgroup of patients may be predictive of the response to treatment or of its preventive effect if the treatment is initiated earlier. The third point is that some effects have been observed on the contralateral knee in these two long-term trials with GlcN.S. The minimum joint space width (JSW) of the contralateral knee was 4.72 ± 1.52 mm, which is similar to the values observed in the general population. The effect of GlcN.S on the contralateral knee was not significantly different from placebo, but there was a trend for prevention of OA (p = 0.17). The fourth point is that the structure modifying effect of GlcN.S was particularly evident in these patients with better-preserved joint space width at baseline, with joint structure closer to that of the general population [46]. The fifth point is that the patients in these two long-term studies were followed for another 5 years after the termination of the 3-year trials. Pooled incidence of total knee replacement during a mean follow up was lower in GlcN.S-treated population than in placebo group (14.5% vs. 6.3 %, p = 0.024) [47]. However, one important limitation of this kind of study is that in the follow up period the patients have been treated in different ways. The debate about possible clinical efficacy is based on two 3-year studies, but recent analysis of 4 structure-modifying trials did not confirm the preventive effect of GlcN.S or GlcN.H [48].

Recently, the EFSA panel of experts has published its scientific position in relation to the substantiation of a health claim related to GlcN and maintenance of joints [49]. The claimed effect was that GlcN “contributes to the protection of joint cartilage exposed to excessive motion or loading and helps to improve the range of motion in joints”. The target population proposed by the applicant was healthy individuals exposed to excessive load on the joint. Maintenance of joints was a beneficial physiological effect. The applicant provided two human studies as pertinent to the claim. The first study was a randomized, double-blind, placebo-controlled trial [50] including 121 male patients who had a recent history of acute knee sport injury. They received either 1.5 g GlcN (n=62) per day or placebo (cellulose; n = 59) for 28 days. Pain and functional ability were evaluated at the beginning of the study and at 7, 14, 21 and 28 days. Supplementation with GlcN demonstrated significant improvement in knee flexion and extension but not in pain and swelling. The EFSA panel of experts considered that the provided evidence did not establish that patients with acute knee injury are representative of the target population with regards to the status of joint tissues or that results obtained in studies on subjects with acute knee injury can be extrapolated to the proposed target population (healthy individuals exposed to excessive load on the joints). In the second human study [51], 21 male soccer players (19–22 years of age, mean 20.3) and as, a control group, 10 male college students (20–27 years of age, mean 23.5) were recruited. They carried out an open label intervention with GlcN.H. To this end, the subjects were divided into two groups that received 1.5 g (n=9) or 3 g (n=10) GlcN per day for three months. Urinary samples were taken at baseline, after the GlcN administration (at three months) and three months after withdrawal of GlcN administration (at six months). The end point of the study involved measurement of the urinary concentrations of CTX-II, C2C, two markers of type II collagen degradation, CPII, a marker of type II collagen synthesis, and the ratio CTX-II/CPII. The administration of 1.5 to 3 mg of GlcN significantly decreased CTX-II levels (p<0.01) [51] but the effect was transient and disappeared after withdrawal of the administration. The panel has noted that this intervention study was not adequately controlled for factors that may have influenced the urinary analysis over the duration of the study, and that the provided evidence did not establish that changes in urinary CTX-II over a period of three months can predict significant changes in type II collagen in joint cartilage. The panel concluded that a cause and effect relationship has not been established between the consumption of GlcN and the maintenance of joint health because of missing appropriate clinical trials in the target (i.e. healthy) population. Besides some evidence for the beneficial effects of GlcN, the remarks of the EFSA outline the limitations of clinical investigations to demonstrate maintenance of joint health.

### Can GlcN reduce the risk of OA development?

The question as to whether GlcN has effectiveness to prevent OA development has not been tested specifically among human subjects. Therefore, it is necessary to consider the processes by which OA develops, and make extrapolations from observations made in animal models and other types of studies about how GlcN might have efficacy in this situation.

With respect to the potential of an intervention as a preventive strategy for OA, it is important to realize that such an effect could be mediated either through an influence on the risk factor profile, or through biological mechanisms that interact directly with pathophysiological processes in OA.

The effects of GlcN on other constitutional characteristics that might represent risk factors for OA (such as body weight) are unlikely or untested, with the possible exception of systemic inflammation. Elevated C-reactive protein levels have been associated with OA [52] and with the progression of hand and wrist OA [53]. The pathophysiological basis of this relationship is uncertain, but it is possible that it reflects the effects of adipose-mediated inflammation on articular structures, or, alternatively, some level of synovitis. In addition, studies during the past 2 years have uncovered diverse pathways that cause chondrocytes to become activated and produce pro-inflammatory mediators, through activation of inflammatory genes regulated by transcription factors such as NF-κB [54]. Furthermore, animal studies [55,56], and one human clinical trial [57], have suggested that the suppression of inflammation may reduce structural progression of OA. It has been proposed that GlcN might be able to intervene in these inflammatory pathways through a number of mechanisms. For example, Imagawa et al. found that O-GlcNAc could prevent cytokine-induced demethylation of a specific CpG site in the IL-1β promoter and this was associated with decreased expression of IL-1β [58]. Other investigators have generated *in vitro* evidence that GlcN.S attenuates NF-κB activation at concentrations comparable to those seen in patients following oral supplemental ingestion [59].

Due to the competition between GlcN and glucose not only at the level of cellular uptake through the GLUT transporters but also in the hexosamine pathway (one of the alternative routes of glucose metabolism), patients treated with this compound may have increased insulin resistance, as suggested by findings in some animals studies, although not in others [42,60,61]. It is therefore important to address the question: “Could oral intake of GlcN interfere with metabolic syndrome and insulin resistance?” With respect to any influence on an individual’s risk factor profile, there is a potential for an adverse interaction of GlcN with aspects of the metabolic syndrome, which is common among elderly people with OA [62]. Effects of GlcN on insulin sensitivity through the hexosamine biosynthetic pathway have been reported in animal models [63]. Indeed, intravenous GlcN was used to produce an animal model for insulin resistance and diabetes as part of early research in that field. It is important to investigate the potential negative side effects of GlcN on glucose metabolism, in particular in diabetic and ‘pre-diabetic’ patients. Several studies have tested this possibility. Studies from Scroggie et al. [64] and Muniyappa et al. [65] showed no effect on insulin resistance after GlcN use, whereas Pham et al. [66] reported data indicating that GlcN worsens insulin sensitivity at doses that are being taken for OA.

In one metabolic study of oral GlcN on serum glucose and insulin levels among 16 patients with OA, three participants demonstrated significant incremental elevations in glucose levels after ingestion of GlcN.S [67]. The authors concluded that GlcN ingestion might affect glucose levels and consequent glucose uptake in patients who have untreated diabetes or glucose intolerance. Dostrovsky et al. recently performed a systematic review to determine if exogenous GlcN adversely affects glucose metabolism in humans [68]. They included 11 studies (6 randomized controlled trials and 5 prospective studies). Four found decreased insulin sensitivity or increased fasting glucose in subjects taking GlcN. They concluded that the available evidence is mixed with regard to the effect of oral GlcN on glucose metabolism, and that more studies are needed, particularly some including subjects at high risk for impairment in glucose homeostasis.

In contrast, high intravenous and intra-arterial doses of GlcN did not affect insulin sensitivity, secretion or action [69,70]. Another study in healthy volunteers that received orally repeated once-daily doses of 1500 mg GlcN.S did not show any changes in serum insulin or blood glucose levels [71]. In turn, patients with Type 2 diabetes receiving a combination of GlcN and chondroitin sulfate for 3 months had no change in their diabetes management or hemoglobin A1c concentrations [64]. Finally, fasting plasma glucose levels were not modified in short-term clinical trials with GlcN.S, as well as in the Reginster long-term trial [1]. In contrast, Pavelka reported that four patients developed diabetes during a 3-year study; however, three of them were in the placebo group and only one was on GlcN.S treatment [2]. A systematic literature review suggests that GlcN has no effect on fasting blood glucose levels, glucose metabolism, or insulin sensitivity at any oral dose level in healthy subjects, individuals with diabetes, or those with impaired glucose tolerance [61]. As concluded in the latter reference, the available evidence implicating GlcN as a diabetogenic agent are limited to rodent infusion studies and *in vitro* observations, experimental models that were determined not to be relevant to humans. Longitudinal studies in large cohorts of healthy human subjects are therefore needed.

Some animal studies and human case reports suggest that chronic use of GlcN in diabetic or “pre-diabetic” individuals might lead to or exacerbate insulin resistance. However the level of proof is not sufficient and we lack long-term studies of GlcN use in individuals with diabetes or pre-diabetes. Based on the available evidence, no specific recommendation for controlling glycemia in patients taking GlcN is proposed.

The existence of the conflicting published reports highlights the paucity of information and the need for further studies in this area.

### Concluding remarks

GlcN is one of the most widely used “over the counter” dietary supplements for the management of OA. In some European countries GlcN is a registered drug. The aim of the Hohenheim Conference and this consensus paper was not to explore one more time the efficacy of GlcN on symptoms and structure in patients with knee or hip OA. This has already been done numerous times in meta-analyses and literature reviews, including a Cochrane review [72,73]. Despite the extensive studies and interest in this compound, its use is still a matter for debate. The aim of this conference was to provide consensual comments on the potential beneficial effect of GlcN on joint health (Tables 1 and 2).

The first question to address was whether normal chondrocytes need GlcN for their physiological function and metabolism. The answer is clearly no because cells favour glucose as the main fuel for energetic production and production of cartilage proteoglycans [24]. In presence of normal glucose concentration, the uptake of GlcN is completely inhibited [74]. Substrate level competition may operate in situations where there is a local decrease in glucose levels or a significant increase in the local concentration of GlcN (millimolar concentrations). Another aspect of the question concerns GlcN and ageing chondrocytes. During the process of chondrocyte senescence, several factors (cell apoptosis, oxidative stress, decreased responsiveness to growth factors, telomere erosion, glycation of ECM macromolecules) contribute to the modification of ECM production in a quantitative and qualitative manner and, as a consequence, this may lead to decreased capacity of the cartilage repair. The lesions observed in ageing cartilage seem similar to those observed in OA cartilage. Thus cartilage ageing is a major risk factor of OA but for many scientists it is still considered to be a different condition to OA. It is therefore important to develop novel tools and assays for mechanistic *in vitro* studies on the effect of GlcN on cartilage ageing. It will be also interesting to see whether GlcN can influence the production of advance glycation end products (AGEs).

The local anti-inflammatory action of GlcN has clearly been demonstrated by many *in vitro* studies [44,58,75-78]. An important consideration of future researches could be more in-depth studies of the systemic and general anti-inflammatory effects of GlcN. This will be interesting in the context of a link between metabolic syndrome and knee OA; it will be indeed important to explore whether GlcN might be able to interfere with metabolic syndrome. One proposal could be to observe the systemic effect of GlcN, for instance on ultrasensitive CRP, a marker of molecular inflammation associated with metabolic syndrome. Improving this molecular inflammatory state in patients with knee OA and metabolic syndrome could be an important target of future studies. This potential general effect of GlcN surely needs to be better explored. A recent study showed that high intake of GlcN in rabbits with both articular and vascular disease, is able to diminish the general inflammation and reduce atheromatous plaques, suggesting that oral GlcN.S may exert systemic anti-inflammatory actions [42].

In the line of a potential systemic effect of GlcN, data regarding the extra articular actions of GlcN, especially on muscle are missing. A recent work shows that in patients suffering from knee OA, GlcN.S reduced pain and improved muscle strength with resistance training in a randomized placebo controlled trial [79]. GlcN.S might also act at distance of the joint, in tissues where its concentration is high such as intestine and liver [38]. This might represent an interesting approach in the future.

How and to whom should we propose any preventive effect of GlcN? Our proposal is to stratify and select some populations without symptomatic OA but submitted to a risk factor, which could potentially be influenced by GlcN. Thus, individuals with a history of traumatic joint injuries (i.e. meniscal tears, anterior cruciate ligament injury) or subjects engaged in high load ballistic sports with repetitive joint impact presenting a higher risk of cartilage degradation may be targeted. In this setting, GlcN may present a beneficial effect on joint structure since we hypothesize that oral GlcN is locally available into the joint and may favour cartilage repair. Similarly, individuals with early OA (doubtful stage 1 in the Kellgren-Lawrence classification system) could be a target for preventive treatment with GlcN. Ageing people without overt OA is also a potentially good population to target. Finally, individuals with metabolic syndrome constitute an interesting population to follow, since GlcN may demonstrate not only local but also systemic effects. One key (but difficult) question is to determine which surrogate biomarkers should be adopted to demonstrate the clinical benefits of GlcN on OA development. In other words, how can we detect small variations in cartilage metabolism that could potentially indicate increased cartilage turnover in normal subjects? So far no such biomarkers have been validated in normal subjects. Assays for several biological molecules have been developed as indicators of cartilage and bone metabolism in OA patients [80]. A recent study comparing soccer players (without knee pain and without radiological OA) to healthy but non-sporty young subjects, has shown that treatment with GlcN for 3 months decreases type II collagen degradation (as measured by urinary CTX-II levels) but GlcN did not affect the increase of type II collagen synthesis (reflected by CP-II serum level) [51]. We propose that a combination of biological markers such as Coll2-1, Coll2-1 NO_2_, COMP, HA or fibuline-3 fragments (Fib3-1 and Fib3-2) should be used as surrogate biomarkers of cartilage function in such studies [4,81]. In addition, sophisticated and new imaging modalities such as MRI with dGemric (delayed gadolinium-enhanced MRI) would be interesting to use in this context for examining the content of biological markers associated with load-bearing (i.e. proteoglycans and their constituent GAGs) in cartilage. The volume of cartilage, the presence and severity of synovitis and/or bone marrow lesions (BML) could also be investigated by MRI. In a recent and relevant study performed in obese patients without symptomatic knee OA (only 32% present radiographic knee OA), it was demonstrated that weight loss has structure modifying effects as expressed by a gain in the medial dGemric index which indirectly reflects the GAG content of cartilage [82]. This kind of study using a surrogate marker such as dGemric index is typically one that could be applied for studying the structure modifying properties of GlcN in patients without overt OA but those exposed to one or more risk factors for developing OA. The use of scintigraphy with specific binding of macrophages may also be interesting in future studies on GlcN [83]. The goal of this kind of study should be to demonstrate that long-term treatment with GlcN may favourably influence some markers of cartilage metabolism, a prerequisite condition that would be indicative but not conclusive of a chondroprotective effect.

The final question concerns the type of GlcN and the dosage of the molecule that should be recommended. GlcN salts are hydrolysed by the acidic pH of the gastric cavity and therefore it is probable that both GlcN.H and GlcN.S result in the same form preceding subsequent intestinal absorption and liver metabolism. A recent paper showed bioequivalence of the two forms. Both in rats and in human volunteers, a single dose charge of GlcN.OH or GlcN.S results in the same levels of GlcN excretion in the urine [84]. Therefore, there is not sufficient data to differentiate between the two forms of GlcN, at least from a pharmacokinetic point of view. Absorption of GlcN after oral administration is limited, leading to low bioavailability (varying between animal species and human).

GlcN remains a controversial molecule; general conclusions and perspectives are to move toward new types of clinical trials in healthy subjects that may be able to explore the potential role of GlcN, considered as a nutriment, to prevent the secondary development of osteoarticular diseases. We have proposed in the present paper some statements and proposals for further studies.

## Abbreviations

AGE: Advance glycation end-product; Cmax: Maximum plasma concentration; ECM: Extracellular matrix; Fib: Fibuline; GAG: Glycosaminoglycan; GAIT: Glucosamine hydrochloride/chondroitin sulfate Arthritis Intervention Trial; GlcN: Glucosamine; GlcN.H: Glucosamine hydrochloride; GlcN.S: Glucosamine sulfate; GLUT: Glucose transporter; HO: Heme oxygenase; iNOS: Inducible nitric oxide synthase; JSN: Joint Space Narrowing; JSW: Joint Space Width; NIH: National Institutes of Health; OA: Osteoarthritis; O-GlcNAc: O-N-acetylglycoaminylation; PGE: Prostaglandin.

## Competing interests

Y Henrotin is the founder of Artialis SA a spin-off of the University of Liège. His research has been supported by educational grant from Bioiberica, Nestle, Royal Canin, Expanscience, Danone and BioXtract. The other authors have no conflict of interest related to the subject of this paper. A. Mobasheri acknowledges the financial support of The Wellcome Trust, the National Centre for the Replacement, Refinement, and Reduction of Animals in Research (NC3Rs; grant number: Mobasheri. A. 28102007), the Biotechnology and Biological Sciences Research Council (BBSRC; grants BBSRC/S/M/2006/13141 and BB/G018030/1) and the Engineering and Physical Sciences Research Council (EPSRC). Y Henrotin, H Weinans and A. Mobasheri are members of the D-BOARD Consortium funded by European Commission Framework 7 Programme (EU FP7) and wish to acknowledge funding from the Commission in relation to project number 305815, (Novel Diagnostics and Biomarkers for Early Identification of Chronic Inflammatory Joint Diseases, HEALTH.2012.2.4.5-2).

## Authors’ contributions

YH chaired the consensus conference, formulated the questions and compiled the answers to the questions. HW and AM addressed question 1. GHB addressed question 2. PK has written the answer to question 3. MT wrote the answer to question 4. XC wrote the concluding remarks. SC reviewed the draft and compiled the consensus remarks and research recommendations. AM and TM edited the final version of the manuscript. HB co-chaired the consensus conference. All authors have read and approved the final manuscript.
